# Clinical Features and Surgical Outcomes of Cats With Presumed Primary Lens Instability: A Retrospective Study of 34 Cases (2018–2022)

**DOI:** 10.1111/vop.70215

**Published:** 2026-06-23

**Authors:** Havi Sarfaty, Raaya Ezra‐Elia, Nili Kahane, Shai Sandalon, Yifat Segev, Lionel Sebbag

**Affiliations:** ^1^ EYECARE Clinic Rinatya Israel; ^2^ Koret School of Veterinary Medicine The Hebrew University of Jerusalem Rehovot Israel

**Keywords:** feline, glaucoma, lens luxation, lens subluxation, ocular surgery, primary lens instability

## Abstract

**Objective:**

Describe the clinical features, disease progression, and outcomes of presumed primary lens instability (PLI) in cats, and to assess its association with glaucoma.

**Animals Studied:**

Thirty‐four cats (68 eyes).

**Procedures:**

Medical records were reviewed for signalment, clinical history, ophthalmic findings, and treatment. Eyes were classified as anterior lens luxation (ALL) or subluxated lens (SLL). Clinical monitoring included intraocular pressure (IOP), menace response, absence/presence of blepharospasm, and complications. Surgical eyes underwent intracapsular lens extraction or phacoemulsification, with or without vitrectomy.

**Results:**

Cats were 1–13 years old (median 3 years) and predominantly male (82%). At presentation, 68% of eyes had ALL and 32% of eyes had SLL. Blepharospasm was observed in 25% of eyes, mostly in ALL. IOP was elevated (> 25 mmHg) in 38% of eyes overall, comprised of 62% SLL eyes (33 ± 5.7 mmHg) and 25% ALL eyes (54.8 ± 24.1 mmHg). Of SLL eyes followed ≥ 90 days, 73% progressed to ALL within a median of 15 days (5–174 days). Forty‐four eyes underwent surgery and, of 42 with follow‐up (median 284 days), 95% achieved comfort and 85% retained vision; however, 70% developed postoperative glaucoma within 68 ± 51 days that was still present in 35% of eyes at last recheck. Retinal complications occurred in 14% of operated eyes. Nonsurgical eyes also showed a high risk for IOP elevation.

**Conclusions:**

Presumed PLI presents bilaterally in generally young cats, often with limited signs of ocular discomfort, but with a high risk of concurrent or delayed glaucoma. Lens extraction restores comfort and can preserve vision but does not prevent future IOP elevation.

## 1 Introduction

Primary lens instability (PLI) is a rare and poorly characterized condition in cats. While PLI in dogs is well documented and linked to specific genetic mutations, reports in cats are scarce, and the underlying cause remains largely unknown. In feline patients, lens displacement is most often considered secondary to chronic uveitis, trauma, glaucoma, or intraocular neoplasia [1], and truly primary cases are likely underrecognized and seldom reported.

A large retrospective study published in 1991 reviewed 345 cases of feline lens luxation collected from a multicenter clinical database. In that series, 2.6% of affected cats exhibited unilateral lens luxation with concurrent subluxation in the fellow eye, and in 28.7% of cases lens displacement was reported in the absence of other overt ocular abnormalities, raising the possibility that not all cases were secondary in nature [2]. Two decades later, Payen et al. reported bilateral PLI in 10 related domestic shorthair cats spanning three generations [3]. Their genetic investigation focused on genes linked to ectopia lentis, including *FBN1*, *ADAMTSL4*, *ADAMTS10*, and *ADAMTS17* [4, 5, 6]. The *ADAMTS17* gene, known to cause PLI in several dog breeds [7], was of particular interest. While their findings implicated an association with a mutation in *FBN1*, a definitive causal relationship could not be established. Since then, no additional published studies have investigated the genetic basis or clinical features of PLI in cats.

Over the past decade, we have observed an increasing number of young cats presenting with bilateral lens instability and no evidence of concurrent ocular or systemic disease. These cases show a consistent clinical pattern suggestive of an unrecognized hereditary condition. The aim of this study was to characterize the clinical features, monitor disease progression, assess surgical and nonsurgical outcomes, and potential associations with glaucoma in cats.

## 2 Materials and Methods

### 2.1 Data Collection

Medical records of cats diagnosed with lens instability at EYECARE clinic (Israel) between January 2018 and December 2022 were reviewed. Cases were included only if lens instability was bilateral and if anamnesis and ophthalmic examination revealed no evidence of trauma, advanced cataracts, chronic uveitis, or chronic glaucoma.

Data collected included breed, sex, age, habitat (indoor or outdoor), duration of clinical signs before presentation, previous treatments prescribed by the referring veterinarian, ocular findings, concurrent systemic diseases, and any known affected family members. Ophthalmic examination findings recorded for each eye included menace response, presence or absence of ocular discomfort, corneal opacities or ulcers, signs of uveitis, cataract severity (if present), lens position, fundoscopic abnormalities, and intraocular pressure (IOP). Ocular discomfort, albeit subjective, was primarily assessed based on the presence/absence of blepharospasm, excessive tearing, or resistance to periocular manipulation. Mild uveitis was characterized by low intraocular pressure, mild iris vascular congestion and/or mild aqueous flare, whereas severe uveitis was associated with more pronounced inflammatory changes. For surgically treated cases, additional data included the surgical technique, use of vitrectomy, the intraoperative medications administered, and the postoperative treatment protocol. Follow‐up evaluations included menace response, fluorescein staining, uveitis signs, fundoscopic findings, IOP, and subsequent interventions.

### 2.2 Ophthalmic Examination

All cats underwent complete ophthalmic examination, including slit‐lamp biomicroscopy (SL‐17, Kowa Company Ltd.), indirect ophthalmoscopy (Vantage Plus Binocular Indirect Ophthalmoscope, Keeler), rebound tonometry (TONOVET Plus, Icare), and fluorescein staining of the ocular surface (AKti‐flu, AKtive Srl). Surgical candidates also received full physical examination and preanesthetic bloodwork, including complete blood count and serum biochemistry.

### 2.3 Treatment

Nonsurgical management involved topical ophthalmic solutions administered 1–3 times daily (case‐dependent), including 2% dorzolamide (Trusopt, Fareva Mirabel), 1% brinzolamide (Azopt, Alcon), 0.1% diclofenac (Voltaren Ophtha, Novartis Consumer Health), and 0.5% tropicamide (Mydramide, Fischer Pharmaceuticals). Systemic acetazolamide (Uramox, Taro Pharmaceutical Industries) at 7 mg/kg twice daily was prescribed in selected feline cases where topical therapy was not feasible due to poor patient compliance.

Surgical lens removal was performed using either intracapsular lens extraction (ICLE) or phacoemulsification using a bimanual (two‐handed) technique [8], with automated anterior vitrectomy performed at the surgeon's discretion when vitreous migration into the anterior chamber occurred; in all phacoemulsification cases, the remaining lens capsule was completely removed.

Intraoperative medications included intracameral adrenaline, triamcinolone, and carbachol as needed. At the end of surgery, all eyes received intracameral injections of 25 μg tissue plasminogen activator (tPA), 0.5 mg moxifloxacin, and 1 mg dexamethasone. Postoperative treatment varied depending on clinical findings and included combinations of systemic antibiotics (amoxicillin/clavulanic acid, cephalexin, doxycycline), systemic anti‐inflammatories (methylprednisolone, meloxicam), and topical medications (chloramphenicol‐polymyxin B, ofloxacin, prednisolone, diclofenac, nepafenac, tropicamide, dorzolamide, and brinzolamide).

### 2.4 Data Analysis

Eyes were categorized by lens position at presentation as anterior lens luxation (ALL) or subluxated lens (SLL). Data were also assessed by treatment modality, that is, surgical group (SG) or nonsurgical group (NSG). Progression from SLL to ALL was recorded for longitudinal assessment. IOP was classified as normal (10–25 mmHg), elevated (> 25 mmHg), or low (< 10 mmHg) [9, 10]. The < 10 mmHg threshold was applied empirically and considered by the authors as supportive of uveitis when accompanied by compatible clinical findings, recognizing that inflammatory signs may be subtle in cats; values between 10 and 15 mmHg were regarded as less specific and interpreted with caution. The estimated onset of IOP elevation or lens progression from SLL to ALL was defined as the midpoint between the last normal and first abnormal examination.

Descriptive statistics were used to summarize demographic and clinical data. Proportions were calculated based on the number of eyes successfully evaluated for each parameter, thereby accounting for variations in patient cooperation and examination feasability.

Clinical outcomes were evaluated for both surgical and nonsurgical groups, focusing on four key parameters: (i) menace response, (ii) IOP, (iii) ocular discomfort, and (iv) postoperative complications, including corneal and retinal events.

## 3 Results

### 3.1 Animals

Thirty‐four domestic shorthair cats (68 eyes) met the inclusion criteria; some had been adopted as young kittens from the street or through rescue organizations, and none were purpose‐bred. There were 28 males (82%; 3 intact, 25 castrated) and 6 females (18%; 2 intact, 4 spayed), aged 1–13 years (median 3 years). Habitat was reported for 32 cats: 19 (59%) indoor‐only, 12 (38%) indoor/outdoor, and one (3%) outdoor‐only. Three cats had a known family history; two were siblings, one with a suspected affected mother.

### 3.2 Clinical Findings

Owners most often reported corneal cloudiness (68%), signs of ocular pain (41%), anisocoria (23%), tearing or discharge (15%), and apparent vision loss (9%). Clinical signs were typically present for an average of 18.8 ± 20.1 days prior to referral, with a median duration of 10 days (range 1–75 days). Eleven cats (32%) had received topical therapy before referral, most commonly prednisolone, dorzolamide, tropicamide, ofloxacin, brinzolamide, or diclofenac. Bloodwork results were available for 24 cats, and 11 of these (46%) showed mild to moderate elevations in serum creatinine (1.45–2.4 mg/dL). Systemic comorbidities were identified in 8 (23%) cats and included chronic asthma, urinary tract disease, heart murmur, suspected chronic kidney disease, and otitis. These findings occurred across a range of ages.

On ophthalmic exam, incidental ocular abnormalities were identified in two cats: one cat had Florida spot keratopathy, and a different cat had persistent pupillary membranes consisting of thin iris‐to‐cornea strands [11, 12]. Lens position varied among the 34 cats: 13 (38%) exhibited bilateral ALL, 20 (59%) had unilateral ALL and contralateral SLL, and one (3%) showed bilateral SLL. Across the 68 eyes examined, 46 (68%) had ALL and 22 (32%) had SLL. Of the 11 SLL eyes monitored for ≥ 90 days, 8 (73%) progressed to ALL within 33.6 ± 57.2 days (median 15 days, range 5–174 days). No clinical evidence of pigment dispersion was observed in the eyes included in this study.

### 3.3 Overall

**FIGURE 1:**
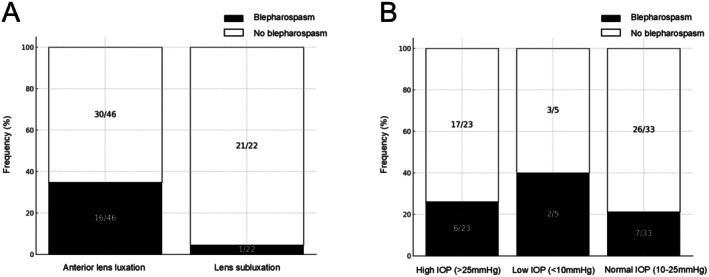
Proportion of feline eyes with (black) and without blepharospasm (white) in relation to (A) lens location and (B) intraocular pressure (IOP) category. IOP was classified as high (> 25 mmHg), normal (10–25 mmHg), or low (< 10 mmHg). Numbers within bars indicate the number of affected eyes out of the total evaluated for each category.

Blepharospasm was present in 17/68 (25%) eyes; 16/17 (94%) were in eyes with ALL. This clinical sign was usually associated with abnormal IOP or the presence of corneal ulcers (Figure 1). Mild anterior uveitis was observed in 5/68 (7%) eyes and was limited to ALL. Menace response was absent in 14/57 (25%) eyes; 4/14 of these (29%) also had high IOP. Across all eyes, IOP was high in 23/61 (38%) eyes (42.5 ± 19.4 mmHg, median 35 mmHg, range 26–99 mmHg), low in 5/61 eyes (8%), and normal in 33/61 (54%) eyes. Additional details are provided in Table S1.

### 3.4 Anterior Lens Luxation

Among eyes with ALL, menace response was present in 26/39 (67%) of those evaluated. Mean ± SD IOP was 24 ± 21.8 mmHg (median 14 mmHg, range 3–99 mmHg), with 10/40 eyes (25%) exhibiting elevated IOP averaging 54.8 ± 24.1 mmHg (median 48.8 mmHg, range 29–99 mmHg). Mild anterior uveitis occurred in 5/46 (11%) eyes, corneal ulceration in 4/46 (9%) eyes, and clinical signs of ocular discomfort in 16/46 (35%) eyes with ALL. Corneal ulcers were characterized as punctate lesions associated with focal areas of dense corneal edema.

### 3.5 Lens Subluxation

**FIGURE 2:**
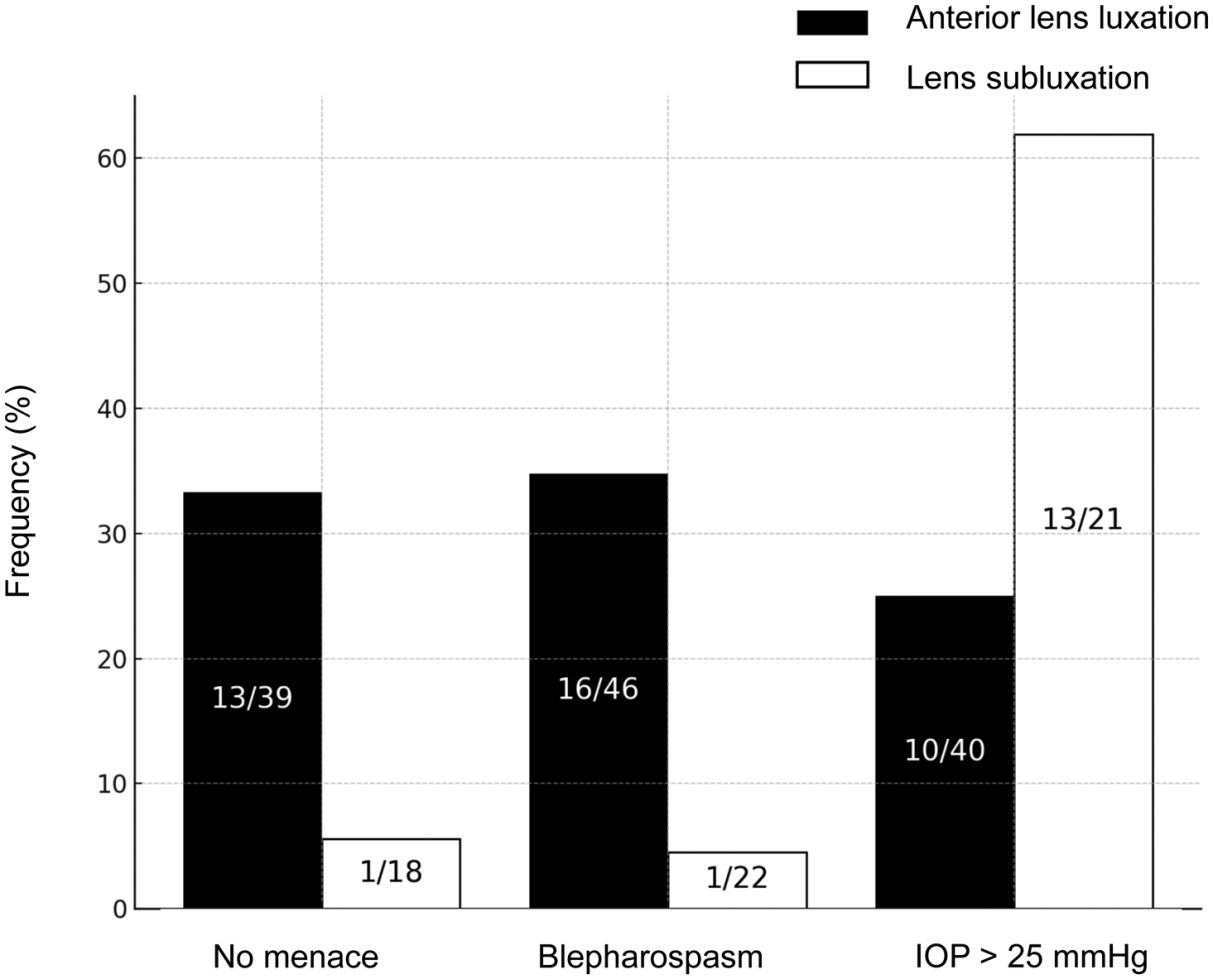
Frequency of absent menace response, blepharospasm, and elevated intraocular pressure (> 25 mmHg) at presentation in cats with anterior lens luxation (black) or lens subluxation (white). Numbers within bars indicate affected eyes out of the total evaluated for each clinical parameter.

In SLL eyes, menace response was positive in 17/18 (94%) eyes evaluated. Mean IOP was 27.9 ± 8.7 mmHg (median 27 mmHg, range 11–43.5 mmHg), and 13/21 (62%) eyes had IOP values above 25 mmHg (mean 33 ± 5.7 mmHg, median 33.3 mmHg, range 26–43.5 mmHg). No eyes showed anterior uveitis and only one eye (1/22, 5%) showed discomfort. Figure 2 provides a summary of menace response, IOP measurements, and blepharospasm for each group.

### 3.6 Treatment and Outcomes

#### 3.6.1 Surgical Group

**FIGURE 3:**
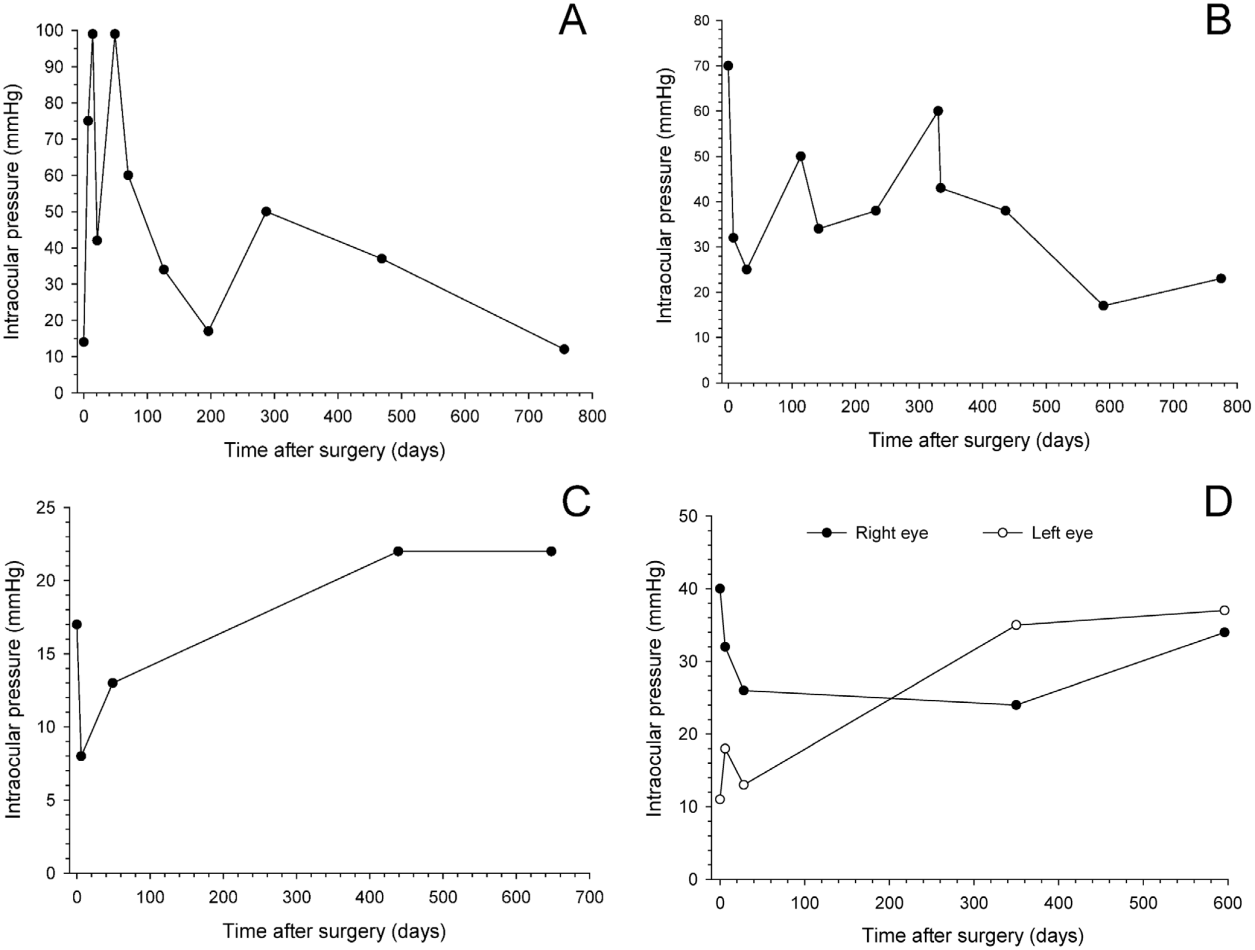
Representative intraocular pressure (IOP) measurement over time in five feline eyes before (time 0) and after lens extraction. (A) Eye with normal preoperative IOP that increased postoperatively then decreased on daily 1% brinzolamide ophthalmic solution. (B) Eye with elevated IOP preoperatively that decreased after surgery and daily 1% brinzolamide ophthalmic solution. (C) Eye with IOP that remained within the normal range without anti‐glaucoma therapy throughout follow‐up. (D) Both eyes of the same cat: The left eye was initially normotensive, and both eyes developed postoperative IOP elevation; no anti‐glaucoma therapy was initiated in either eye owing to poor patient compliance.

A total of 44 eyes underwent surgery, including 32 ALL, 5 SLL, and 7 that progressed to ALL during follow‐up. All five subluxated lenses were removed concurrently with the fellow eye undergoing surgery for anterior luxation, with no apparent zonular traction or ciliary body hemorrhage observed intraoperatively. The unstable lens was removed via ICLE in 32 eyes and phacoemulsification in 12 eyes, while anterior vitrectomy was performed in 36 eyes. Two cats lacked follow‐up data and were excluded from determination of treatment outcome. Among 42 eyes with follow‐up (324 ± 248 days, median 284, range 10–775), corneal ulceration occurred in one eye within 30 days, while retinal complications developed in 6 eyes (14%) within 205 ± 287 days (median 91 days, range 14–775 days). Retinal complications included retinal detachment (*n* = 3; following ICLE), retinal atrophy (*n* = 2; all phacoemulsification), and focal intraretinal hemorrhage (*n* = 1; ICLE). At the last follow‐up, 40/42 (95%) appeared comfortable without blepharospasm or signs of active uveitis, while 32/37 (85%) appeared visual based on an intact menace response. However, 28/40 (70%) eyes developed elevated IOP in at least one follow‐up exam, first detected at 68 ± 51 days postoperatively (median 84 days, range 1–322 days), as detailed in Table 1 and Figure 3. Postoperative glaucoma was managed with transscleral cyclophotocoagulation in two eyes, maintaining stable IOP for up to 1423 days postoperatively, and globe removal in three cases (10 to 1005 days postoperatively). Despite therapy, glaucoma was still present in 14/40 (35%) eyes at the last recheck exam. Table 1 provides additional information about blepharospasm, menace response and IOP at the last follow‐up examination, in relation to preoperative status. Further details are provided in Table S2 and Table S3.

**TABLE 1:** Summary of menace response, intraocular pressure, and clinical signs of discomfort and uveitis before and after surgical treatment. Outcomes are presented for all eyes combined and by surgical technique [intracapsular lens extraction (ICLE) and phacoemulsification (phaco)] at the final recheck (42 eyes).

Menace response	All (*n* = 36)	11.1% (*n* = 4): Negative before, gained vision	72.2% (*n* = 26): Positive before, remained positive	5.6% (*n* = 2): Negative before, remained negative	11.1% (*n* = 4): Positive before, lost vision
	ICLE (*n* = 27)	11.1% (*n* = 3): Negative before, gained vision	66.7% (*n* = 18): Positive before, remained positive	7.4% (*n* = 2): Negative before, remained negative	14.8% (*n* = 4): Positive before, lost vision
	Phaco (*n* = 9)	11.1% (*n* = 1):Negative before, gained vision	88.9% (*n* = 8): Positive before, remained positive		
Intraocular pressure	All (*n* = 40)	25% (*n* = 10): High before, decreased	40% (*n* = 16): Normal before, remained normal	10% (*n* = 4): High before, remained high	25% (*n* = 10): Normal before, increased to high
	ICLE (*n* = 29)	20.7% (*n* = 6): High before, decreased	38% (*n* = 11): Normal before, remained normal	10.3% (*n* = 3): High before, remained high	31% (*n* = 9): Normal before, increased to high
	Phaco (*n* = 11)	36.4% (*n* = 4): High before, decreased	45.4% (*n* = 5): Normal before, remained normal	9.1% (*n* = 1): High before, remained high	9.1% (*n* = 1): Normal before, increased to high
Discomfort	All (*n* = 42)	95% (*n* = 40): Eyes comfortable			5% (*n* = 2): Showed signs of discomfort
	ICLE (*n* = 31)	93.5% (*n* = 29): Eyes comfortable			6.5% (*n* = 2): Showed signs of discomfort
	Phaco (*n* = 11)	100% (*n* = 11): Eyes comfortable			

#### 3.6.2 Nonsurgical Group

Twenty‐four eyes were managed nonsurgically (14 with ALL and 10 with SLL) but only 7 eyes (2 ALL, 5 SLL) had sufficient follow‐up of 127 ± 131 days (median 116 days, range 11–373 days). None of the SLL eyes in the nonsurgical group progressed to ALL during the follow‐up period. One eye lost menace response, two developed discomfort, and four developed IOP elevations exceeding 25 mmHg.

## 4 Discussion

This study describes a cohort of cats with lens instability suspected to be primary in origin. The clinical presentation differed from the more typical secondary lens luxation patterns encountered in cats, which are often unilateral and associated with chronic ocular disease, such as uveitis,cataract, or glaucoma [2, 13, 14, 15, 16]. In veterinary medicine, lens luxation in cats is most often regarded as secondary to these conditions. The first report suggesting a familial, likely genetic origin of lens luxation in cats was published in 2011 by Payen et al. [3]. However, an earlier retrospective study of 345 feline cases by Olivero et al. noted that 28.7% of affected cats had no other ocular signs [2], implying that primary or hereditary lens luxation may have existed but was historically under‐recognized and under‐investigated. Notably, this large multicenter study by Olivero et al. provided valuable epidemiologic insights but relied on heterogeneous clinical records collected across multiple institutions, with limited standardization of ophthalmic examination and glaucoma assessment. In contrast, the present study reflects a smaller, single‐center cohort with more uniform clinical evaluation and follow‐up, allowing for more detailed characterization of lens instability and associated complications. These differences highlight the complementary nature of the two approaches and emphasize the importance of interpreting findings within the context of the study design.

Assessment of ocular pain in cats is inherently challenging, and the absence of overt clinical signs does not necessarily equate to true ocular comfort [17]. In this cohort, however, pain was not a prominent clinical feature. Discomfort was primarily observed in the small number of eyes with concurrent corneal ulceration. In these cases, ulceration was typically associated with focal areas of corneal edema rather than diffuse corneal involvement, consistent with a localized corneal injury rather than widespread corneal disease. Most cats exhibited minimal signs of ocular discomfort, even in the presence of elevated intraocular pressure. Consistent with our findings, Payen et al. also identified minimal ocular pain as a characteristic feature of feline PLI [3]. This clinical picture contrasts with canine anterior lens luxation, which in the authors' experience is often painful even with modest IOP elevations. A plausible explanation may be the relatively deeper anterior chamber in cats compared to dogs, allowing a luxated lens to remain in the anterior chamber without consistent contact with the iris or cornea. In addition, the vertically slit feline pupil may be less susceptible to obstruction by a luxated lens or prolapsed vitreous than the round canine pupil, potentially reducing the likelihood of pupillary block.

A striking finding in this study was the high prevalence of elevated IOP in eyes with SLL, with 62% of eyes exceeding 25 mmHg (mean 33 mmHg). Data on IOP changes in dogs with lens subluxation are limited, but the rate observed here appears unusually high. Mechanical irritation from intermittent contact with the posterior iris could theoretically release pigment, triggering inflammatory obstruction of aqueous outflow [18]. Alternatively, zonular rupture may allow vitreous prolapse through the pupil or iridocorneal angle, creating a pupillary or angle block [13]. Here, the absence of vitreous strands and the rarity of uveitis in our cases suggest that other factors contribute to IOP elevations. A concomitant genetic susceptibility to impaired aqueous humor outflow or primary glaucomatous change cannot be excluded and may reflect an inherited predisposition contributing to the observed IOP elevations in these cats. However, the exact mechanism underlying the high prevalence of IOP elevation in feline eyes with ALL or SLL remains uncertain and could not be determined in this retrospective study.

Interestingly, eyes with complete ALL showed elevated IOP less frequently, occurring in only 25% of cases, despite higher mean IOP (54.8 mmHg). The reason for this paradox is uncertain. Possible contributing factors include mild uveitis induced by anterior luxation, which can transiently lower IOP, as well as reduced forward displacement of the iris compared to subluxation, where the partially unstable lens may push the iris forward and promote iridocorneal angle closure. In comparison, canine studies report glaucoma in 45%–77.8% of anterior luxation cases [19, 20, 21, 22], likely reflecting species differences in anterior chamber depth and susceptibility to pupillary block. Further, in contrast to dogs, feline patients in the present study exhibited minimal pre‐ and postoperative uveitis, suggesting that inflammation was unlikely to be the primary driver of IOP elevation.

IOP measurements in eyes with lens instability should be interpreted with caution. Given the retrospective nature of this study, it cannot be stated with certainty that all measurements were consistently obtained from corneal regions entirely free of focal edema or disease. However, it is routine practice in our clinic to avoid areas of corneal pathology during tonometry and, in eyes with anterior lens luxation, to obtain measurements as far as possible from the region of lens displacement. Nevertheless, corneal edema and altered anterior segment anatomy have been shown to influence tonometric readings and may result in inaccurate measurements [23, 24].

While preexisting elevation in IOP or glaucoma cannot be entirely excluded in this retrospective study, we consider this possibility unlikely. Eyes in which lens displacement was accompanied by elevated intraocular pressure did not exhibit clinical features typically associated with chronic glaucoma, such as globe enlargement, optic nerve or retinal atrophy, or corneal edema, nor were there findings consistent with long‐standing uveitis. The absence of such structural indicators argues against a prolonged glaucomatous process sufficient to cause gradual zonular breakdown and secondary lens luxation. Accordingly, these findings are more compatible with a primary lens instability process, while acknowledging that definitive temporal relationships between glaucoma and lens displacement cannot be established with certainty.

All 32 eyes with ALL in this study underwent lens extraction surgery. The clinical rationale included reduction of IOP in cases where it was elevated at diagnosis, prevention of future IOP elevation, and pain relief in painful eyes. Prevention of progressive endothelial disease was not a primary surgical consideration, except in the few eyes with corneal edema where reducing the risk of secondary ulceration was taken into account. Prevention of secondary cataract development was not a consideration at this stage. Importantly, postoperative glaucoma developed in many cases despite surgery, often weeks to months later. This delayed onset, together with similar findings in nonsurgical eyes, suggests an underlying glaucoma predisposition rather than a purely surgical or inflammatory complication. These results parallel observations in dogs, where Glover et al. reported 34% postoperative glaucoma after intracapsular lens extraction [22]. Given the high frequency of postoperative IOP elevation observed, concurrent prophylactic glaucoma surgery (e.g., endoscopic cyclophotocoagulation, transscleral cyclophotocoagulation, or gonio‐implant placement) may be considered at the time of lens extraction, although their role in cats with presumed primary lens instability requires further evaluation.

Retinal detachment occurred in only 7% of eyes, all from ICLE, lower than the 37.5% (6/16) reported in dogs with ALL that underwent lens extraction [22]. This difference may reflect species‐specific anatomy, reduced postoperative inflammation in cats, or both.

Seven eyes with anterior luxation were not surgically treated, either due to owner refusal when the eye appeared comfortable and normotensive, or following the authors' early experience when it became apparent that surgery did not consistently prevent later IOP elevation. Consequently, in later cases, surgery was generally reserved for eyes exhibiting discomfort. Follow‐up was limited, but four of seven eyes developed elevated IOP within 3 months, reinforcing the importance of regular monitoring even in clinically quiet eyes. Unlike in dogs with subluxation, which are often treated prophylactically with miotics such as latanoprost, latanoprost has not been specifically evaluated for prevention of anterior lens luxation in cats. In one experimental study, topical latanoprost induced marked but transient miosis, with rebound mydriasis observed approximately 24 h after administration, and the miotic response diminished with continued twice‐daily treatment [25]. These findings suggest that latanoprost may not provide sustained, long‐term miosis in cats, thereby limiting its theoretical utility for preventing anterior lens migration. Future studies may help determine whether miotic therapy could delay progression from SLL to ALL in some cases. Cases of subluxation or confirmed instability should be closely monitored by the owner and veterinarian, and anterior lens migration should prompt a treatment decision.

From a genetic perspective, feline PLI shares features with human ectopia lentis, a rare disorder affecting 6.4 per 100 000 individuals [26]. In humans, the most common cause is a mutation in the *FBN1* gene on chromosome 15, which also causes Marfan syndrome and is associated with systemic abnormalities [4]. Other implicated genes include *ADAMTSL4* [5], *ADAMTS10* and *ADAMTS17*, which are also responsible for hereditary lens luxation in dogs [6]. In a group of 10 genetically related cats diagnosed with PLI, mutations in *FBN1, ADAMTS17*, *ADAMTSL4*, and *ADAMTS10* were ruled out [3]. The *LTBP2* gene is of particular interest in microfibrils structure [27]. The *LTBP2* mutation, first identified in 2016 as a cause of congenital glaucoma in cats [28], impairs development by disrupting the morphogenesis of a specific TM subpopulation [29]. Affected cats often exhibit lens instability, but early globe enlargement makes it difficult to determine whether this is primary or secondary [30]. The original study focused on TM pathology and did not assess zonular or ciliary body abnormalities. Ranocchia et al. evaluated the same *LTBP2* variant previously implicated in feline glaucoma and excluded it as a cause of primary glaucoma in Australian Burmese cats; however, other variants within *LTBP2* or mutations in different genes cannot be excluded, consistent with the genetic heterogeneity of glaucoma in cats [31]. In the present study, formal pedigree relationships could not be established among affected cats, many of which were adopted as strays. Future studies using genomic approaches, including whole genome sequencing, may help clarify relatedness and identify genetic factors associated with lens instability.

In conclusion, we describe an apparently high prevalence of presumed primary lens instability in cats from a relatively restricted geographical location, a condition that typically presents bilaterally in young animals. Ocular pain was not a prominent clinical feature in this cohort, despite a high prevalence of elevated intraocular pressure; however, in the subset of eyes where ocular discomfort was present preoperatively, surgical lens extraction resulted in resolution of discomfort and may help preserve vision. Postoperative glaucoma was common and often delayed, suggesting an underlying heritable glaucoma predisposition, rather than solely mechanical or inflammatory mechanisms for secondary glaucoma. Nonsurgical management is feasible in comfortable eyes, but both surgical and nonsurgical cases require lifelong IOP monitoring due to the high risk of glaucoma. These findings emphasize the need for careful clinical surveillance and support further genetic investigations to clarify the pathophysiology and familial basis of feline primary lens instability.

## Author Contributions

**Raaya Ezra‐Elia:** methodology, writing – original draft, writing – review and editing, project administration, formal analysis, conceptualization. **Yifat Segev:** data curation, resources, methodology. **Havi Sarfaty:** conceptualization, supervision, writing – original draft, writing – review and editing, project administration, methodology, data curation, resources. **Nili Kahane:** data curation, resources, methodology. **Lionel Sebbag:** conceptualization, supervision, writing – original draft, writing – review and editing, methodology, data curation, resources. **Shai Sandalon:** data curation, resources, methodology.

## Disclosure

Artificial Intelligence Statement: The authors have nothing to report.

## Ethics Statement

This study complies with the Guidelines for Ethics Research in Veterinary Ophthalmology (GERVO) and is exempt from approval by an ethics committee.

## Supporting information


**Table S1:** Clinical findings at presentation in 68 eyes of 34 cats with presumed primary lens instability. IOP, Intraocular pressure.
**Table S2:** Clinical outcomes at the final follow‐up examination in 44 surgically treated eyes from 25 cats with presumed primary lens instability. Abbreviations: IOP, intraocular pressure; TSCPC, transscleral cyclophotocoagulation.
**Table S3:** Longitudinal intraocular pressure (mmHg) measurements following surgical lens extraction in 44 eyes from 25 cats with presumed primary lens instability.

## Data Availability

The data that support the findings of this study are available from the corresponding author upon reasonable request.
