# Supplementary material for: Clinical Features and Surgical Outcomes of Cats With Presumed Primary Lens Instability: A Retrospective Study of 34 Cases (2018–2022)
Source: Vet Ophthalmol. 2026 Jun 23;29(4):e70215. doi: 10.1111/vop.70215 (PMC13291341; doi:10.1111/vop.70215)
Supplement: Supplementary file 1 — Table S1: Clinical findings at presentation in 68 eyes of 34 cats with presumed primary lens instability. IOP, Intraocular pressure. Table S2: Clinical outcomes at the final follow‐up examination in 44 surgically treated eyes from 25 cats with presumed primary lens instability. Abbreviations: IOP, intraocular pressure; TSCPC, transscleral cyclophotocoagulation. Table S3: Longitudinal intraocular pressure (mmHg) measurements following surgical lens extraction in 44 eyes from 25 cats with presumed primary lens instability. [file VOP-29-0-s001.docx]

**Supplementary Table 1.** Clinical findings at presentation in 68 eyes of 34 cats with presumed primary lens instability. IOP = Intraocular pressure.

| **Cat ID** | **Eye** | **Lens location** | **Menace** | **Blepharospasm** | **IOP (mmHg)** | **Surgery** |
| --- | --- | --- | --- | --- | --- | --- |
| 1 | Left | Subluxated | Yes | No | 11 | No |
| 1 | Right | Anterior luxation | No | Yes | 22 | Yes |
| 2 | Left | Subluxated | Yes | No | 43 | Yes |
| 2 | Right | Anterior luxation | Yes | No | 11 | Yes |
| 3 | Left | Subluxated | – | No | 27 | No |
| 3 | Right | Anterior luxation | – | No | 28 | Yes |
| 4 | Left | Subluxated | Yes | No | 34 | No |
| 4 | Right | Subluxated | No | No | 14 | No |
| 5 | Left | Anterior luxation | No | Yes | 10 | Yes |
| 5 | Right | Anterior luxation | No | No | 13 | No |
| 6 | Left | Anterior luxation | Yes | No | – | Yes |
| 6 | Right | Subluxated | Yes | No | – | No |
| 7 | Left | Anterior luxation | No | No | 14 | No |
| 7 | Right | Anterior luxation | No | Yes | 14 | Yes |
| 8 | Left | Anterior luxation | Yes | No | 14 | Yes |
| 8 | Right | Anterior luxation | Yes | No | 14 | Yes |
| 9 | Left | Subluxated | Yes | No | 28 | Yes |
| 9 | Right | Anterior luxation | Yes | No | 13 | Yes |
| 10 | Left | Anterior luxation | No | Yes | 75 | No |
| 10 | Right | Subluxated | Yes | No | 28 | No |
| 11 | Left | Anterior luxation | Yes | No | 24 | No |
| 11 | Right | Anterior luxation | Yes | No | 10 | No |
| 12 | Left | Subluxated | – | No | 24 | Yes |
| 12 | Right | Anterior luxation | – | No | 17 | Yes |
| 13 | Left | Anterior luxation | – | Yes | 7 | Yes |
| 13 | Right | Subluxated | – | No | 24 | Yes |
| 14 | Left | Anterior luxation | Yes | Yes | 37 | Yes |
| 14 | Right | Subluxated | Yes | No | 32 | Yes |
| 15 | Left | Anterior luxation | Yes | No | 7 | No |
| 15 | Right | Anterior luxation | Yes | No | 7 | No |
| 16 | Left | Anterior luxation | Yes | Yes | 22 | Yes |
| 16 | Right | Subluxated | Yes | No | 26 | Yes |
| 17 | Left | Anterior luxation | Yes | No | 75 | Yes |
| 17 | Right | Anterior luxation | Yes | No | 25 | Yes |

**Supplementary Table 1** (continued).

| **Cat ID** | **Eye** | **Lens location** | **Menace** | **Blepharospasm** | **IOP (mmHg)** | **Surgery** |
| --- | --- | --- | --- | --- | --- | --- |
| 18 | Left | Anterior luxation | Yes | No | 70 | Yes |
| 18 | Right | Subluxated | Yes | No | 35 | Yes |
| 19 | Left | Subluxated | Yes | No | 13 | Yes |
| 19 | Right | Anterior luxation | – | No | 15 | Yes |
| 20 | Left | Anterior luxation | Yes | Yes | 21 | Yes |
| 20 | Right | Subluxated | Yes | Yes | 33 | Yes |
| 21 | Left | Anterior luxation | Yes | Yes | 30 | Yes |
| 21 | Right | Anterior luxation | Yes | Yes | 13 | Yes |
| 22 | Left | Anterior luxation | Inconsistent | No | 14 | Yes |
| 22 | Right | Anterior luxation | No | No | 7 | Yes |
| 23 | Left | Subluxated | Yes | No | 23 | Yes |
| 23 | Right | Anterior luxation | Yes | No | 11 | Yes |
| 24 | Left | Anterior luxation | Yes | No | 16 | Yes |
| 24 | Right | Subluxated | Yes | No | 27 | Yes |
| 25 | Left | Anterior luxation | Yes | No | 11 | Yes |
| 25 | Right | Subluxated | Yes | No | 40 | Yes |
| 26 | Left | Anterior luxation | No | Yes | 3 | Yes |
| 26 | Right | Anterior luxation | No | Yes | 13 | Yes |
| 27 | Left | Subluxated | – | No | 40 | No |
| 27 | Right | Anterior luxation | – | No | – | Yes |
| 28 | Left | Subluxated | Yes | No | 22 | No |
| 28 | Right | Anterior luxation | No | No | 13 | No |
| 29 | Left | Anterior luxation | No | Yes | 99 | No |
| 29 | Right | Subluxated | Yes | No | 25 | No |
| 30 | Left | Anterior luxation | Yes | No | 14 | Yes |
| 30 | Right | Anterior luxation | Yes | No | 17 | Yes |
| 31 | Left | Anterior luxation | – | Yes | – | No |
| 31 | Right | Anterior luxation | – | Yes | – | No |
| 32 | Left | Anterior luxation | Yes | No | – | No |
| 32 | Right | Anterior luxation | Yes | No | – | No |
| 33 | Left | Anterior luxation | Inconsistent | No | 40 | Yes |
| 33 | Right | Anterior luxation | No | No | 35 | Yes |
| 34 | Left | Subluxated | Yes | No | 35 | No |
| 34 | Right | Anterior luxation | No | Yes | 57 | No |

**Supplementary Table 2.** Clinical outcomes at the final follow-up examination in 44 surgically treated eyes from 25 cats with presumed primary lens instability. Abbreviations: IOP, intraocular pressure; TSCPC, transscleral cyclophotocoagulation.

| Cat ID | Eye | Pre-operative status | | | Last follow-up visit | | | | | |
| --- | --- | --- | --- | --- | --- | --- | --- | --- | --- | --- |
|  |  | Menace | Blepharospasm | IOP (mmHg) | Days | Menace | Blepharospasm | IOP (mmHg) | Retinal status | Additional data |
| 1 | Right | No | Yes | 22 | 13 | No | No | 6 |  |  |
| 2 | Left | Yes | No | 43 | 126 | Yes | No | 37 |  | TSCPC  1423 days post-op |
| 2 | Right | Yes | No | 11 | 126 | Yes | No | 37 |  | TSCPC  1423 days post-op |
| 3 | Right | – | No | 28 | – | – | – | – |  |  |
| 5 | Left | No | Yes | 10 | 105 | Yes | No | 22 |  |  |
| 6 | Left | Yes | No | – | 30 | Yes | Yes | 14 |  |  |
| 7 | Right | – | No | 14 | – | – | – | – |  |  |
| 8 | Left | Yes | No | 14 | 287 | Yes | No | 30 |  |  |
| 8 | Right | Yes | No | 14 | 287 | Yes | No | 32 |  |  |
| 9 | Left | Yes | No | 28 | 61 | Yes | No | 11 |  |  |
| 9 | Right | Yes | No | 13 | 61 | Yes | No | 16 |  |  |
| 12 | Left | – | No | 24 | 636 | Yes | No | 22 |  |  |
| 12 | Right | – | No | 17 | 648 | Yes | No | 22 |  |  |
| 13 | Left | – | Yes | 7 | 30 | – | No | 9 |  |  |
| 13 | Right | – | No | 24 | 10 | – | Yes | 80 |  | Enucleation  10 days post-op |
| 14 | Left | Yes | Yes | 37 | 289 | Yes | No | 32 |  |  |
| 14 | Right | Yes | No | 32 | 281 | Yes | No | 37 |  |  |
| 16 | Left | Yes | Yes | 22 | 722 | Yes | No | 16 |  |  |
| 16 | Right | Yes | No | 26 | 680 | Yes | No | 15 |  |  |
| 17 | Left | Yes | No | 75 | 91 | Yes | No | 14 |  |  |
| 17 | Right | Yes | No | 25 | 337 | Yes | No | 24 | Detachment |  |

**Supplementary Table 2** (continued).

| Cat ID | Eye | Pre-operative status | | | Last follow-up visit | | | | | |
| --- | --- | --- | --- | --- | --- | --- | --- | --- | --- | --- |
|  |  | Menace | Blepharospasm | IOP (mmHg) | Days | Menace | Blepharospasm | IOP (mmHg) | Retinal status | Additional data |
| 18 | Left | Yes | No | 70 | 775 | No | No | 23 | Atrophy | Enucleation  1005 days post-op |
| 18 | Right | Yes | No | 35 | 775 | No | No | 40 | Atrophy | Enucleation  1005 days post-op |
| 19 | Left | Yes | No | 13 | 14 | Yes | No | 15 | Focal bleeding |  |
| 19 | Right | Yes | No | 15 | 19 | Yes | No | 12 |  |  |
| 20 | Left | Yes | Yes | 21 | 161 | Yes | No | 20 |  |  |
| 20 | Right | Yes | Yes | 33 | 161 | No | No | 20 | Detachment |  |
| 21 | Left | Yes | Yes | 30 | 169 | Yes | No | 19 |  |  |
| 21 | Right | Yes | Yes | 13 | 169 | Yes | No | 22 |  |  |
| 22 | Left | Inconsistent | No | 14 | 756 | Inconsistent | No | 12 |  |  |
| 22 | Right | No | No | 7 | 756 | Yes | No | 14 |  |  |
| 23 | Left | Yes | No | 23 | 501 | Yes | No | 26 |  |  |
| 23 | Right | Yes | No | 11 | 501 | Yes | No | 26 |  |  |
| 24 | Left | Yes | No | 16 | 516 | Yes | No | 31 |  |  |
| 24 | Right | Yes | No | 27 | 352 | Yes | No | 33 |  |  |
| 25 | Left | Yes | No | 11 | 596 | Yes | No | 37 |  |  |
| 25 | Right | Yes | No | 40 | 596 | Yes | No | 34 |  |  |
| 26 | Left | No | Yes | 3 | 239 | Yes | No | 13 | Detachment |  |
| 26 | Right | No | Yes | 13 | 239 | Yes | No | 12 |  |  |
| 27 | Right | – | No | – | 349 | Yes | No | 27 |  |  |
| 30 | Left | Yes | No | 14 | 328 | Yes | No | 26 |  |  |
| 30 | Right | Yes | No | 17 | 303 | Yes | No | 31 |  |  |
| 33 | Left | Inconsistent | No | 40 | 6 | No | No | 10 |  |  |
| 33 | Right | No | No | 35 | 264 | No | No | – |  |  |

**Supplementary Table 3.** Longitudinal intraocular pressure (mmHg) measurements following surgical lens extraction in 44 eyes from 25 cats with presumed primary lens instability.

| Cat ID | Eye | Pre-operative | 1-14 d | 15-30 d | 1-3 months | | | | 3-6 months | | 6-12 months | | | | | 12-24 months | | > 24 months |
| --- | --- | --- | --- | --- | --- | --- | --- | --- | --- | --- | --- | --- | --- | --- | --- | --- | --- | --- |
| 1 | Right | 22 | 5 | 6 | – | – | – | – | – | – | – | – | – | – | – | – | – | – |
| 2 | Left | 43 | 13 | – | 36 | 22 | 23 | 28 | 37 | – | – | – | – | – | – | – | – | – |
| 2 | Right | 11 | 13 | – | 38 | 23 | 32 | 37 | 37 | – | – | – | – | – | – | – | – | – |
| 3 | Right | 28 | – | – | – | – | – | – | – | – | – | – | – | – | – | – | – | – |
| 5 | Left | 10 | 17 | – | 17 | – | – | – | 22 | – | – | – | – | – | – | – | – | – |
| 6 | Left | – | 5 | 14 | – | – | – | – | – | – | – | – | – | – | – | – | – | – |
| 7 | Right | 14 | - | – | – | – | – | – | – | – | – | – | – | – | – | – | – | – |
| 8 | Left | 14 | 10 | 12 | – | – | – | – | 25 | – | 30 | – | – | – | – | – | – | – |
| 8 | Right | 14 | 13 | 14 | – | – | – | – | 21 | – | 32 | – | – | – | – | – | – | – |
| 9 | Left | 28 | – | – | 7 | 11 | – | – | – | – | – | – | – | – | – | – | – | – |
| 9 | Right | 13 | – | – | 10 | 16 | – | – | – | – | – | – | – | – | – | – | – | – |
| 12 | Left | 24 | – | – | 8 | – | – | – | – | – | – | – | – | – | – | 26 | 22 | – |
| 12 | Right | 17 | 8 | – | 13 | – | – | – | – | – | – | – | – | – | – | 22 | 22 | – |
| 13 | Left | 7 | 99 | 20 | 9 | – | – | – | – | – | – | – | – | – | – | – | – | – |
| 13 | Right | 24 | 67 | 80 | – | – | – | – | – | – | – | – | – | – | – | – | – | – |
| 14 | Left | 37 | 10 | 16 | – | – | – | – | – | – | 32 | – | – | – | – | – | – | – |
| 14 | Right | 32 | 8 | 16 | – | – | – | – | – | – | 37 | – | – | – | – | – | – | – |
| 16 | Left | 22 | – | – | 19 | 13 | – | – | – | – | 57 | 40 | 28 | – | – | 21 | 16 | – |
| 16 | Right | 26 | – | 15 | – | – | – | – | – | – | 57 | 60 | 38 | 27 | – | 15 | – | – |
| 17 | Left | 75 | 9 | – | 22 | 50 | – | – | 14 | – | – | – | – | – | – | – | – | – |
| 17 | Right | 25 | 13 | 12 | 27 | – | – | – | – | – | 29 | 45 | 21 | 32 | 24 | – | – | – |
| 18 | Left | 70 | 32 | 25 | – | – | – | – | 50 | 34 | 38 | 60 | 43 | – | – | 38 | 17 | 23 |
| 18 | Right | 35 | 47 | 23 | – | – | – | – | 50 | 33 | 38 | 60 | 45 | – | – | 47 | 45 | 40 |

**Supplementary Table 3** (continued).

| Cat ID | Eye | Pre-operative | 1-14 d | 15-30 d | 1-3 months | | | | 3-6 months | | 6-12 months | | | | | 12-24 months | | > 24 months |
| --- | --- | --- | --- | --- | --- | --- | --- | --- | --- | --- | --- | --- | --- | --- | --- | --- | --- | --- |
| 19 | Left | 13 | 99 | 15 | – | – | – | – | – | – | – | – | – | – | – | – | – | – |
| 19 | Right | 15 | 10 | 12 | – | – | – | – | – | – | – | – | – | – | – | – | – | – |
| 20 | Left | 21 | 9.5 | 16 | 13 | 50 | – | – | 47 | 20 | – | – | – | – | – | – | – | – |
| 20 | Right | 33 | 14 | 0 | 6 | 14 | – | – | 25 | 20 | – | – | – | – | – | – | – | – |
| 21 | Left | 30 | 0 | 12 | 9.5 | 13 | 20 | – | 25 | 19 | – | – | – | – | – | – | – | – |
| 21 | Right | 13 | 11 | 14 | 15 | 22 | 60 | – | 40 | 22 | – | – | – | – | – | – | – | – |
| 22 | Left | 14 | 75 | 99 | 42 | 99 | 60 | – | 34 | – | 17 | 50 | – | – | – | 37 | – | 12 |
| 22 | Right | 7 | 57 | 99 | 21 | 40 | 32 | – | 22 | – | 22 | 47 | – | – | – | 15 | – | 14 |
| 23 | Left | 23 | 16 | 14 | 14 | – | – | – | 18 | – | 24 | – | – | – | – | 26 | – | – |
| 23 | Right | 11 | 16 | 11 | 20 | – | – | – | 23 | – | 23 | – | – | – | – | 26 | – | – |
| 24 | Left | 16 | – | – | – | – | – | – | – | – | 29 | 24 | 23 | – | – | 31 | – | – |
| 24 | Right | 27 | – | 49 | 27 | – | – | – | – | – | 22 | 33 |  | – | – | – | – | – |
| 25 | Left | 11 | 18 | 13 | – | – | – | – | – | – | 35 | – | – | – | – | 37 | – | – |
| 25 | Right | 40 | 32 | 26 | – | – | – | – | – | – | 24 | – | – | – | – | 34 | – | – |
| 26 | Left | 3 | 7 | 5 | 14 | – | – | – | – | – | 13 | – | – | – | – | – | – | – |
| 26 | Right | 13 | 5 | 8 | 13 | – | – | – | – | – | 12 | – | – | – | – | – | – | – |
| 27 | Right | – | – | 18 | 21 | – | – | – | 18 | – | 27 | – | – | – | – | – | – | – |
| 30 | Left | 14 | 13 | 11 | 10 | 11 | – | – | 20 | – | 27 | 26 | – | – | – | – | – | – |
| 30 | Right | 17 | 6 | – | – | – | – | – | – | – | 47 | 31 | – | – | – | – | – | – |
| 33 | Left | 40 | 10 | – | – | – | – | – | – | – | – | – | – | – | – | – | – | – |
| 33 | Right | 35 | 5 | – | – | – | – | – | – | – | 50 | – | – | – | – | – | – | – |
